# Arguments to Support a Viral Origin of Oral Squamous Cell Carcinoma in Non-Smoker and Non-Drinker Patients

**DOI:** 10.3389/fonc.2020.00822

**Published:** 2020-05-21

**Authors:** Jean-Philippe Foy, Chloé Bertolus, David Boutolleau, Henri Agut, Antoine Gessain, Zdenko Herceg, Pierre Saintigny

**Affiliations:** ^1^Univ Lyon, Université Claude Bernard Lyon 1, INSERM 1052, CNRS 5286, Centre Léon Bérard, Centre de Recherche en Cancérologie de Lyon, Lyon, France; ^2^Department of Translational Research and Innovation, Centre Léon Bérard, Lyon, France; ^3^Sorbonne Université, AP-HP, Groupe Hospitalier Pitié-Salpêtrière Charles Foix, Department of Oral and Maxillo-Facial Surgery, Paris, France; ^4^Sorbonne Université, AP-HP, Groupe Hospitalier Pitié-Salpêtrière-Charles Foix, Centre National de Référence Herpèsvirus, Department of Virology, Paris, France; ^5^Sorbonne Université, CR7, Centre d'Immunologie et de Maladies Infectieuses (CIMI-Paris), INSERM U1135, Paris, France; ^6^Department of Virology, Institut Pasteur, CNRS, UMR 3569, Paris, France; ^7^Epigenetics Group, International Agency for Research on Cancer (IARC), Lyon, France; ^8^Department of Medical Oncology, Centre Léon Bérard, Lyon, France

**Keywords:** herpesvirus, HSV-2, EBV, oral squamous cell carcinoma, non-smoker non-drinker, hit and run

## Abstract

In some western countries, an increasing incidence of oral squamous cell carcinoma (OSCC) has been observed in non-smoker non-drinker patients (NSND), mostly in women with HPV-negative OSCC. In the context of the unknown etiology and mechanisms of tumorigenesis of OSCC in NSND, we discuss data supporting the hypothesis of a viral origin not related to HPV. OSCC from NSND are characterized by an antiviral DNA methylation and gene expression signature. Based on the similar increasing incidence of oral tongue SCC (OTSCC) and oropharyngeal SCC (OPSCC) in young women and men respectively, we hypothesize that changes in sexual behaviors may lead to an increasing incidence of herpesvirus in the oral cavity, especially HSV-2, similarly to what has already been described in HPV-positive OPSCC. Because viral genome integration has not been detected in OSCC from NSND, a “hit and run” viral mechanism involving epigenome deregulation could therefore play a key role at early steps of oral carcinogenesis in this population of patients. In conclusion, epidemiological, clinical and molecular data supports a “hit and run” viral origin of OSCC from NSND.

## Introduction

Head and neck cancer (HNC) is ranked as the seventh most frequent cancer worldwide and is a significant cause of cancer-associated morbidity and mortality (1). A squamous origin is more common in HNC which is strongly associated with consumption of tobacco and alcohol. However, HNC is a heterogeneous disease which can be caused by alternative etiological factors, especially viral infection, and which includes different anatomical subsites: oral cavity, oropharynx, nasopharynx, hypopharynx and larynx. Squamous cell carcinomas (SCC) of the larynx and hypopharynx are strongly associated with smoking and drinking habits while nasopharyngeal SCC as well as some oropharyngeal SCC are caused by a viral infection involving Epstein - Barr virus (EBV) and Human Papillomavirus (HPV), respectively. Although alcohol and tobacco still remain associated with oral cavity and oropharyngeal cancers, the incidence of oropharyngeal and oral SCC is increasing in young patients, suggesting alternative etiological factors (2). Notably, an increasing incidence of HNSCC has been observed in young to middle-aged men with oropharyngeal SCC (OPSCC) (3) and has been associated with HPV infection (4, 5). HNSCC can also affect the oral cavity of non-smoker non-drinker (NSND) patients, especially in young and elderly women with oral tongue SCC (OTSCC) and gingival SCC respectively (6–8). Indeed, although some epidemiological studies may provide inconsistent data on the incidence of OSCC worldwide, a previous study using data from 22 international cancer registries showed that the increasing incidence of oral tongue SCC was reported among subjects <45 years old in some countries (9). However, as opposed to HPV-related OPSCC, a direct oncogenic role of HPV during oral carcinogenesis has not been demonstrated (10–12) and therefore, has not been associated with the similar increasing incidence of OTSCC in young women (2, 11). This contrasts with recent reports by us and others showing that OSCC affecting NSND are characterized by gene expression profiles compatible with an antiviral response (13, 14). Herein, we review and discuss the arguments to support the hypothesis of a viral origin of OSCC in NSND alternative to HPV, based on epidemiological, clinical as well as molecular data.

## Increasing Incidence of OSCC in Young NSND: Heredity or Extrinsic Risk Factors?

### Heredity

Constitutional genetic abnormalities may also potentially explain the occurrence of OSCC in young NSND (2). Previously published genetic variations, such as gene polymorphisms (15, 16), have been associated with OSCC but further investigations are required in order to provide conclusive evidence of their relation with smoking and/or drinking habits or not. Moreover, the recent increase of OSCC in young NSND is unlikely to be due to changes in the genetic makeup considering the timescale needed for the acquisitions of new genetic polymorphisms.

Notably, further studies are needed in order to investigate the role of predisposing genetic disorders such as Fanconi and dyskeratosis congenita, into oral carcinogenesis not related to tobacco/alcohol consumption.

### Extrinsic Risk Factors

Extrinsic environmental risk factors contribute significantly to most common cancers (17). In particular, the International Agency for Research on Cancer (IARC) estimated that ~12% of cancers are caused by one of seven known oncogenic viruses: hepatitis B and C viruses (HBV, HCV); human T-lymphotropic virus 1 (HTLV-1); HPV; Kaposi's sarcoma-associated herpes virus (HHV-8); Merkel cell polyomavirus; and Epstein-Barr virus (EBV). In the field of HNSCC, EBV and HPV are involved in nasopharyngeal SCC as well as in OPSCC (4, 5) respectively. Although a comprehensive RNA-sequencing for viral pathogen discovery failed to reveal any potentially causative integrated viruses in OSCC from NSND, the involvement of a non-integrated virus in the initiation or promotion of OSCC cannot be excluded (18). Moreover, additional risk factors may potentially contribute to oral carcinogenesis in NSND. In particular, chronic local inflammation, as observed in periodontal diseases, may induce changes in oral microbiota that would increase the risk of oral cancer (19–21). Interestingly, the reported relationship between HPV status and oral microbiota (22) suggests that an increasing incidence of viral infection in the oral cavity may also interfere with the oral microbial flora and therefore, may contribute to oral cancer.

Moreover, alternative etiological factors, such as drug consumption (23), household air pollution (24), occupation (25), have been proposed to explain oral cancer not related to tobacco/alcohol habits, but require validation in large prospective cohorts of patients. In contrast, some factors, such as the regular consumption of fruits and vegetables could play a role in reducing oral cancer risk (26). While the link between these factors and oral cancer development is not definitive, they may interact with oncogenic viruses and therefore play a possible role in NSND developing OSCC.

In conclusion, a comprehensive epidemiological analysis of the geographical distribution of OSCC in NSND, which is still poorly known worldwide, could help to provide a better understanding of the potential role of genetic predisposition factors as well as the involvement of specific infectious agents in this disease.

## Antiviral Gene Expression and DNA Methylation Profiles Suggest a Viral Origin of OSCC in NSND

### Antiviral Gene Expression Profile

**Figure 1:**
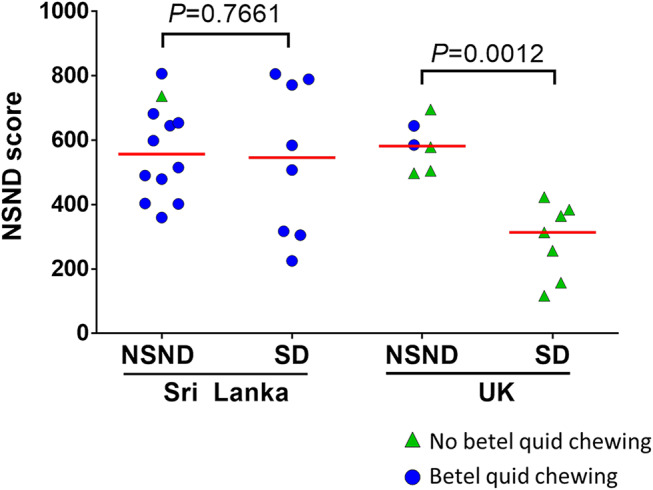
Enrichment scores for the non-smoker non-drinker (NSND) gene set in patients with OSCC from Sri Lankan and United Kingdom (UK). We previously identified a set of genes that were differentially expressed between NSND and smoker drinker patients (14). Using the single sample Gene Set Enrichment Analysis, we computed a score of this gene set (NSND score) in a publically available gene expression dataset of OSCC from Sri Lankan and UK patients (GSE51010) (30).

Because NSND suffering from OSCC are epidemiologically different from SD, their molecular profiles have been compared in different studies (7, 14, 18, 27–29), as summarized in Table 1. Few actionable genomic alterations were different between OSCC from NSND and SD. We compared genome wide expression profiles of HPV-negative OSCC from NSND and SD and found that the main biological difference lies in the differential expression of the genes involved in the immune microenvironment (14). Notably, OSCC from NSND were characterized by an enrichment of immune-related pathways involving T-cell activation and differentiation, which was consistent with a higher CD8+ T-cell infiltrate in NSND compared to SD. Moreover, we observed an activation of the interferon-γ response in OSCC from NSND, as previously reported in all HNSCC from NSND (29). Our data highlighted the importance of the microenvironment, and also suggested a higher clinical benefit of indoleamine 2, 3-dioxygénase (IDO1) and programmed death-ligand 1 (PD-L1) inhibition in the subgroup of NSND patients with OSCC. Notably, we identified a set of ~850 genes that were differentially expressed in NSND vs. SD (named NSND gene set), that were validated in four independent datasets: including one cohort of patients treated at our institution (Centre Léon Bérard, CLB, Lyon, France). Using the same methodology (single sample gene set enrichment analysis) and as shown in Figure 1, we have computed the enrichment score of the NSND gene set in OSCC surgically resected in patients from United Kingdom or from Sri Lanka (GSE51010) (30). In line with our previous results, OSCC from NSND had a higher score compared to OSCC from SD in patients from the United Kingdom. No significant difference was found between NSND and SD patients from Sri Lanka who are betel consumers (Figure 1). This observation provides some evidence that the differences in the immune microenvironment between NSND and SD Caucasian patients could be explained by an extrinsic stimulation of the immune response in NSND, similarly to what has been previously described in OSCC from Sri Lankan betel consumers (30), rather than a negative effect of smoking and drinking on the immune response. This is also in line with the observation that no difference in the enrichment score of the NSND gene set in normal oral mucosa from smokers vs. non-smokers was observed, as well as in normal oral keratinocytes treated with ethanol and/or nicotine vs. untreated ones (14). Overall, these results provide some evidence that the difference in OSCC affecting NSND vs. SD patients are not related to the effect of smoking and/or alcohol on normal mucosa.

**Table 1:** Overview of studies investigating differences in molecular profiles of oral/head and neck squamous cell carcinomas in non-smoker (non-drinker) compared to smoker (drinker) patient.

**PMID**	**First Author (Reference)**	**Year**	**Journal**	**Title**	**Compared groups**	**Tumor site**	**Molecular profiling**	Conclusion* / results in NS(ND) compared to S(D)
10522920	Koch et al. (7)	1999	The Laryngoscope	Head and Neck Cancer in Nonsmokers: A Distinct Clinical and Molecular Entity	NS vs. S	Head Neck	LOH Mutations	• ↓ LOH 3p/4q/11q • ↓ TP53 Mut.
22035108	Farshadpour et al. (29)	2012	Oral Diseases	A gene expression profile for non-smoking and non-drinking patients with head and neck cancer.	NSND vs. SD	Head Neck	mRNA	• ↑IFN-γ • ↓ NFκB
24874835	Pickering et al. (27)	2014	Clinical Cancer Research	Squamous cell carcinoma of the oral tongue in young non-smokers is genomically similar to tumors in older smokers	Young NS vs. old S	Oral Tongue	CNA Mutations	• Overall similarity
24954188	Li et al. (18)	2015	Head and Neck	Clinical, genomic, and metagenomic characterization of oral tongue squamous cell carcinoma in patients who do not smoke	NS vs. S	Oral Tongue	Mutations Meta-genomic	• ↓ TP53 Mut. • No integrated viral sequence was detected
26544609	Kolokythas et al. (28)	2015	Plos One	Similar Squamous Cell Carcinoma Epithelium microRNA Expression in Non Smokers and Ever Smokers	NS vs. S	Oral cavity	miRNA	• Overall similarity of miRNA expression
28460011	Foy et al. (14)	2017	Annals of Oncology	The immune microenvironment of HPV-negative oral squamous cell carcinoma from non-smokers and non-drinkers patients suggests higher clinical benefit of IDO1 and PD1/PD-L1 blockade	NSND vs. SD	Oral cavity	CNAs mutations mRNA	• ↓ mutational load and % CNA • ↓ 11q13 amplification • ↑IFN-γ • ↑ T-cell infiltrate • ↑ PD-L1 • ↑ IDO1

NS, non-smoker; S, smoker, NSND, non-smoker non-drinker; SD, smoker drinker; CNA, copy number alterations; LOH, loss of heterozygosity. *Studies which concluded that NS (ND) were overall similar or different compared to S (D) are highlighted in blue and red respectively.

**Figure 2:**
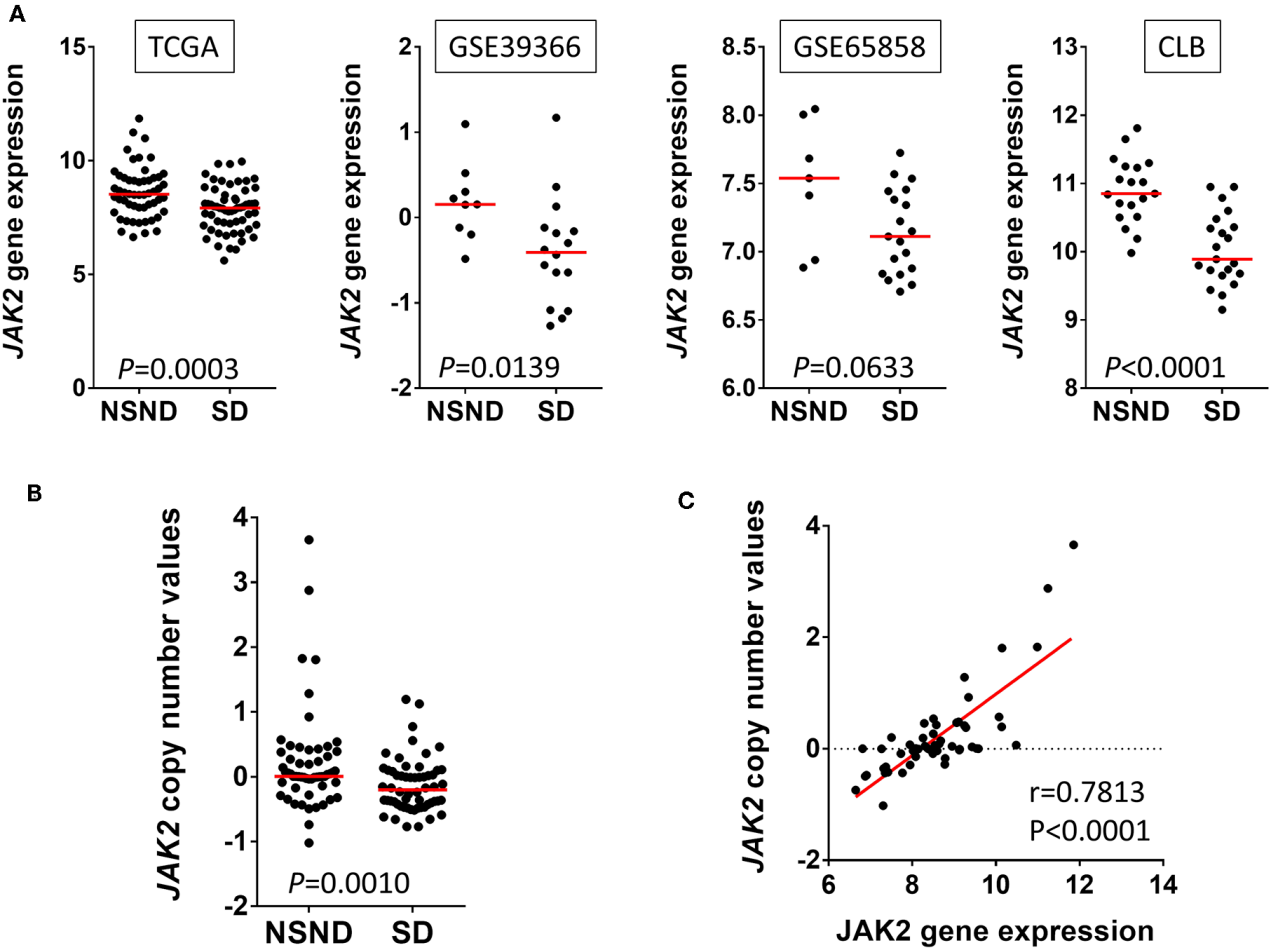
Janus kinase 2 (*JAK2*) alterations in human papillomavirus (HPV)-negative oral squamous cell carcinoma (OSCC) from non-smokers non-drinkers (NSND) and smokers drinkers (SD). **(A)** We extracted *JAK2* gene expression data from four independent cohorts of HPV-negative OSCC, as previously defined (14): The Cancer Genome Atlas (TCGA), GEO1 and GEO2 from GSE39366 and GSE65858 respectively, and Centre Léon Bérard (CLB, Lyon, France). Overexpression of JAK2 in NSND as compared with SD was observed in all four cohorts (Whitney test). In TCGA, we compared copy number linear value in OSCC from NSND and SD (Mann Whitney test) **(B)** and tested its correlation with gene expression in OSCC from NSND (Pearson correlation) **(C)**.

Interestingly, *JAK2* was among the genes overexpressed in NSND compared to SD, in multiple cohorts (Figure 2A). Moreover, *JAK2* copy number gain was observed in some tumors from NSND (Figure 2B), and was correlated with its gene expression level (Figure 2C). *JAK2* is involved in the gamma (or type II) interferon activation in response to intracellular pathogens, including viruses. Intriguingly, concurrent overexpression and amplification of *JAK2* has recently been found in three other cancers: a subgroup of gastric cancers (31), Hodgkin lymphoma, and a subgroup of triple negative breast cancer (32, 33), all being related to EBV infection. It is tempting to hypothesize that gene and expression level alterations of *JAK2* reflect the chronic inflammatory response induced by an infectious agent such a viral infection. Moreover, the lower mutational load in OSCC from NSND compared to SD suggests that the potent IFN-γ-mediated immune response is independent from the mutation load and linked to a dominant tumor antigen, as observed in the case of viral-induced carcinogenesis such as in HPV-related OPSCC (14). Overall, these observations suggest the potential role of chronic-viral stimulation during oral carcinogenesis in NSND. However, caution should be made regarding these data, because of the lack of specific signature of viral infection.

### Antiviral DNA Methylation Profile

A recent study has investigated DNA methylation subtypes of HNSCC, and has identified a CpG Island Methylator Phenotype (CIMP)-atypical subtype of HNSCC more commonly affecting the oral cavity of non-smoker and female patients. Consistently with our results, this subtype was characterized by an antiviral gene expression profile associated with pro-inflammatory M1 macrophages and CD8+ T cell infiltration (13). Viral-associated epigenetic changes are known to be associated with cancer development (32, 33), and CIMP has already been associated with EBV and HPV associated carcinogenesis (34, 35). Upregulation of DNA-methyltransferases in virus-infected cells may lead to aberrant DNA methylation (36) as well as miRNA deregulation such as downregulation of the tumor suppressor miR-200 as observed in EBV-associated gastric adenocarcinomas (37). Thus, DNA methylation profile of OSCC in NSND strengthens the hypothesis of their viral origin.

## Increasing Incidence of Oropharyngeal SCC and OSCC in Young NSND: a Common Epidemiological Origin?

**Figure 3:**
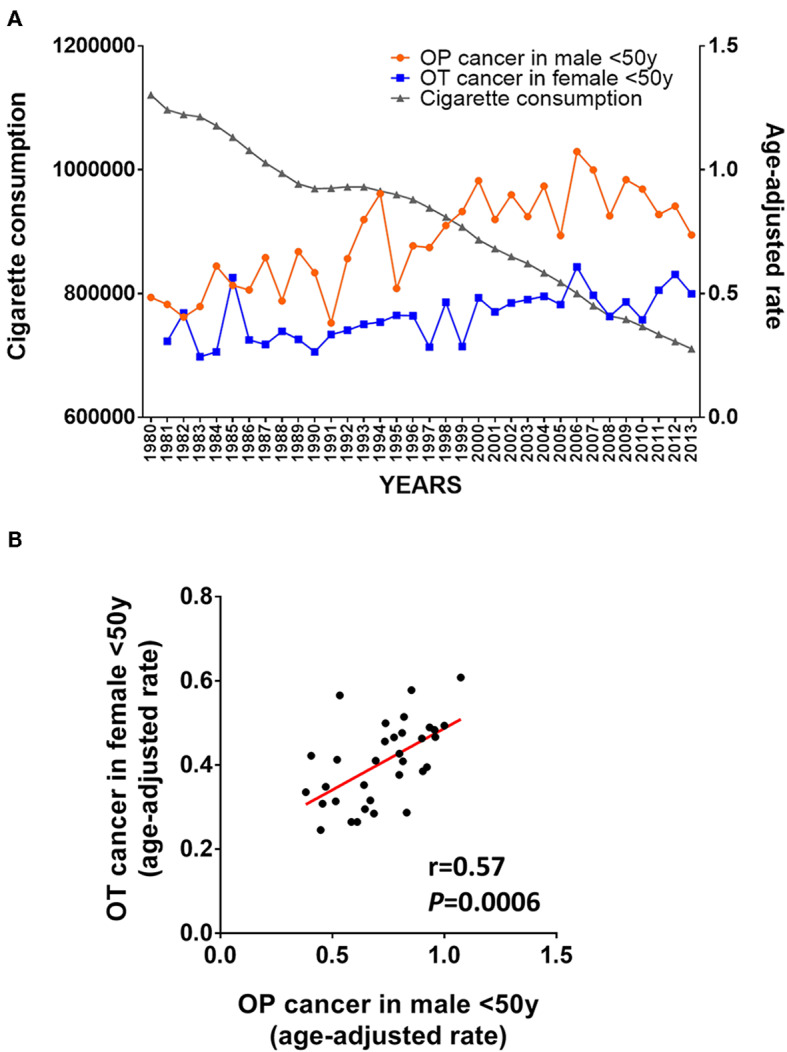
Epidemiological argument supporting a viral origin of OSCC in non-smoker non-drinker (NSND). **(A)** Evolution of age-adjusted cancer incidence in young patients (<50 years-old) from the SEER database (41) and global cigarette consumption between 1980 and 2013 from the Regional Office for the Americas (AMRO) as previously reported (42); **(B)** Pearson's correlation of the age-adjusted incidence of OT and OP cancer in young female and male between 1980 and 2013.

From an epidemiological perspective, an intriguing observation over the past three decades has been the simultaneous increasing incidence of OPSCC in young men as well as OSCC in young women (11, 38–40). In order to illustrate these observations, we mined the SEER database and compared the incidence of OT cancer in women and of OP cancer in men less than 50 years old (41). As shown in Figure 3A, a parallel increase of the incidence of OT cancer in female and of OP cancer in males less that 50 was observed between 1980 and 2013. We contrasted this increase with the decrease in cigarette consumption during the same period of time based on data provided by Ng et al. (42). Consistently, Figure 3B shows a Pearson correlation of 0.57 (*P* = 0.0006) between the incidence of OT cancer in women and of OP cancer in men <50 years old. This observation is consistent with a previous study showing that the increasing incidence of both OTSCC and OPSCC had simultaneously started in the 1980s (43).

The increasing incidence of OPSCC in young NSND men has been associated with an increasing rate of oral HPV infection due to oral sexual behaviors (4, 44). Markedly, the decrease in age of sexual debut as well as the increased number of sexual partners may have contributed to a rise in oral/oropharyngeal HPV infection (44, 45), and a higher risk of HPV transmission from women to men has been proposed to explain the higher incidence of OPSCC in men compared to women (46).

Based on these observations, we propose that the increasing incidence of OTSCC in young NSND may also be linked to one or several sexual transmissible virus which could be transmitted by oral sexual behaviors and which could promote oral carcinogenesis. If this is true, because the increasing incidence of OSCC from NSND is mainly observed in women, transmission of the potential oncogenic virus(es) should be higher from men to women rather than from women to men.

## Viral Oncogenesis of OSCC in NSND: Should We Incriminate Only One Specific Virus?

Molecular profile and epidemiological data on OSCC from NSND support the hypothesis of a viral origin possibly due to changes in sexual behaviors (44, 45). As previously described, viral infections are common among humans, but rarely lead to cancer (47). Epidemiological criteria for causality, proposed by Austin Bradford Hill, have been used in order to provide some evidence for a causal relationship between viral infection and cancer. A total of nine criteria were described, including the strength and consistency of association as well as experimental verification and analogy (47). In order to show the strength and consistency of the association between virus(es) and OSCC in NSND, one of them and/or its viral sequence should be detected in all tumor cells, in different studies by independent investigators. However, using three separate transcriptomic analyses, no viral sequence was found to be integrated in the tumor genome of 20 OSCC from non-smokers (18). In the TCGA cohort of our previous study (14), information on the presence of integrated viral sequences was retrieved for 22 OSCC in NSND, from a previous publication (48). DNA virus transcript was detected in only one OSCC which harbored a human herpes virus type 1. These results do not support the hypothesis of a viral integration-driven oral carcinogenesis in NSND, although it cannot be definitively excluded with regard to the small number of samples as well as the methodology of these studies. A mechanism that has been proposed by which carcinogenesis can be promoted by a viral infection without host genome integration is the “hit and run” phenomenon (18). It has been defined by the International Agency for Research on Cancer as the involvement of a virus in the initiation or promotion of cancer without being required for the maintenance of the transformed phenotype (49). It has been originally described in order to explain the oncogenic potential of herpesviruses, especially herpes simplex virus 2 (HSV-2), in the late 1970s (50) and the early 1980s (51). Further evidence of the role of the “hit and run” mechanism in oncogenesis was provided for adenovirus in the early 2000s (52), and also discussed for HPV in HNSCC (53) or gamma herpesviruses, especially EBV (54). However, a definitive experimental proof of the hit-and-run phenomenon as a mechanism of carcinogenesis is still lacking.

Herpesviruses are extremely widespread among humans and represent a large family of DNA viruses. More than 90% of adults have been infected with at least one of these, and a latent form of the virus remains in most people. There are 9 herpesviruses types known to infect humans: herpes simplex viruses 1 and 2 (HSV-1 and HSV-2), varicella-zoster virus (VZV), Epstein–Barr virus (EBV), human cytomegalovirus (CMV), human herpesvirus 6A and 6B (HHV-6A and HHV-6B), human herpesvirus 7 (HHV-7), and Kaposi's sarcoma-associated herpesvirus (HHV-8). A majority of them is transmitted by saliva, and is characterized by the latent and recurring infections. It is plausible that specific herpesviruses are involved in oral carcinogenesis, through a hit and run mechanism, when affecting individuals with a specific genetic context. In particular, oral shedding HSV-2 has been previously reported (55, 56) and changes in oral sexual behaviors (44, 45) could lead to an increasing incidence of oral infection by HSV-2 which is usually found in genital areas, but related evidence-based literature is scarce. Interestingly, HSV-2 infection has been described as more easily transmitted from men to women than from women to men (57), that would be in line with the increasing incidence of OSCC mostly observed in women, as discussed in the previous section. Moreover, the predilection for HPV-positive HNSCC to occur in palatine and lingual tonsils has been associated with local features such as anatomy of reticular crypt epithelium as well as the local lymphoid microenvironment of the oropharynx (53, 58). Similarly, in order to explain the predilection for OSCC in NSND to occur in the oral cavity, the neurotropism of herpes simplex viruses such as HSV-2 may be associated with the rich innervation (sensitive, sensory/gustation and motor) of the oral cavity involving different cranial nerves.

Besides the involvement of one specific virus in oral carcinogenesis, changes in sexual behaviors (44, 45) may also cause oral transmission of several different genital viruses as well as other microbial species, resulting in changes in the normal microbial flora of the oral cavity. Indeed, a viral-mediated deregulation of the oral microbiome has been already observed in HPV-positive HNSCC which is characterized by a different taxonomic composition of the microbiota in saliva, compared to the HPV-negative disease (22). Such changes in oral microbiome may be associated with chronic inflammation such as in periodontitis and lead to malignant transformation of the oral mucosa (19–21). A recent study has also suggested an association between periodontal pathogens and OSCC in NSND (59).

Finally, an intriguing observation is the difference in antiviral immune response between male and female in terms of prevalence, intensity and pathogenesis of viral infection. Indeed, higher innate and adaptive immune responses in female may contribute to an increased risk of immunopathology due to aberrant host inflammatory responses (60). Bringing this notion to oncogenesis may also explain the higher incidence of this cancer in female compared to male.

## Bridge the Gap Between Herpesviruses, the Hit and Run Hypothesis and Oral Cavity Carcinogenesis in NSND

The evidence suggesting a causal link between herpesvirus infection and the development of OSCC is circumstantial. While the support for this relationship may grow with new studies, the molecular mechanism by which herpesviruses may trigger oral carcinogenesis is largely unknown. We propose that herpesviruses promote oral carcinogenesis, especially in NSND, through a direct, yet to be explored, hit and run mechanism involving epigenome deregulation. The epigenome plays the pivotal role in the establishment and stable propagation of gene activity states over cell generations. Epigenetic mechanisms have been implicated in modulating the gene expression programme in response to environmental exposures, including biological agents, namely viruses (61, 62). In addition, epigenetic modifications are known to play important roles in protecting against viral infection, and there is evidence that some viruses may hijack cellular machineries to promote the viral life cycle (61, 63). Therefore, epigenome reconfiguration, potentially involving DNA methylation and non-coding RNAs, and consequently gene expression reprogramming, may be induced by a transient infection by herpesvirus (“hit”) and these changes may be propagated over many cell division even after the clearance of the virus (“run”). Herpesvirus may hijack cellular defense systems by directly interacting with epigenetic (DNA methylation) machinery, thereby deregulating different key host genes and pathways via an epigenetic strategy, a scenario analogous to that suggested for different oncogenic viruses (32, 61, 64). Several factors related to the DNA methylation process, including DNA methyltransferases (DNMTs) and DNA demethylases (TET enzymes), may be involved in this process. An alternative, albeit not mutually exclusive, mechanism by which herpesvirus may deregulate the epigenome states and gene expression program operating in hit and run oncogenesis in oral mucosa, may involve interaction between viral proteins and host proteins involved in regulation of transcriptional programme, such as transcription factors. This may notably concern a subset of transcription factors (known as “pioneer transcription factors”) which have the capacity to modulate epigenetic states, through opening chromatin and inducing nucleosome remodeling, eviction or affecting DNA-nucleosome interaction thereby making an unscheduled DNA accessibility to other transcriptional factors (65). Another possibility is that the proteins encoded by herpesvirus may interact with cellular proteins that protect the genome from aberrant epigenetic modifications (such as aberrant DNA methylation) that may stably silence or upregulate the key cellular genes involved in the control of cell proliferation and genomic stability.

Identifying and characterizing potential epigenome deregulation in the herpesvirus-mediated hit and run oncogenesis should contribute to understanding the mechanism of oral carcinogenesis. This in turn should serve as the basis for development of methylation-based biomarkers that may be used for follow-up of individuals at higher risk and preventive strategies aimed at reversing early key molecular events leading to cancer development.

## Conclusion

**Figure 4:**
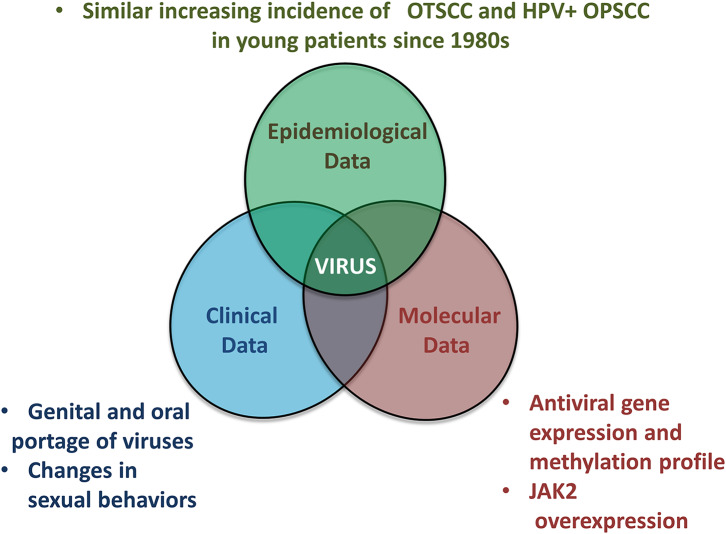
Overview of epidemiological, clinical and molecular evidence linking OSCC affecting non-smokers non-drinkers to a viral origin.

In conclusion, we propose an overview of epidemiological, clinical and molecular data which are in favor of the viral origin of OSCC in NSND (Figure 4). Markedly, OSCC from NSND are characterized by an antiviral DNA methylation and gene expression signature. Because viral genome integration has not been detected in OSCC from NSND, a “hit and run” viral mechanism involving epigenome deregulation could therefore play a key role at early steps of oral carcinogenesis in this population of patients. Based on the similar increasing incidence of OTSCC and OPSCC in young women and men respectively, we hypothesize that changes in sexual behaviors may lead to an increasing incidence of herpesvirus in the oral cavity, especially HSV-2, similarly to what has already been described in HPV-positive OPSCC.

## Data Availability Statement

Publicly available datasets were analyzed in this study. This data can be found here: The Cancer Genome Atlas; Gene expression Omnibus (GSE51010).

## Author Contributions

J-PF, ZH, and PS devised the conceptual ideas and drafted the original manuscript. J-PF performed the literature search and draw the figures. CB, DB, HA, and AG contributed to review and revisions of the final draft. All authors approved the final version of the manuscript.

## Conflict of Interest

The authors declare that the research was conducted in the absence of any commercial or financial relationships that could be construed as a potential conflict of interest.
